# High Temperature Stress Impairs Muscle Quality in Largemouth Bass (*Micropterus salmoides*) Through Textural Deterioration and Flavor Compounds Depletion

**DOI:** 10.3390/biology15080634

**Published:** 2026-04-17

**Authors:** Wanjie Cai, Hui You, Meiyu Wang, Yanjian Jin, Zhiyong Dong, Bo Shi, Yuexing Zhang, Liying Huang

**Affiliations:** 1 National Engineering Research Center for Marine Aquaculture, Marine Science and Technology College, Zhejiang Ocean University, Zhoushan 316022, China; 2 Zhejiang Marine Ecology and Environment Monitoring Center, Zhoushan 316022, China; 3 Department of Animal and Aquaculture Science, Faculty of Bioscience, Norwegian University of Life Science, NO-1432 Ås, Norway; 4 Zhejiang Marine Fisheries Research Institute, Zhoushan 316021, China

**Keywords:** thermal stress, flesh quality, texture, flavor, *Micropterus salmoides*

## Abstract

While the detrimental effects of high temperature stress on fish growth and disease resistance have been widely reported, its impact on muscle quality has received limited attention. In this study, largemouth bass *Micropterus salmoides* with an initial body weight of 45.73 g were subjected to a 60-day growth trial (~25 °C), followed by a 5-day acute warming phase and a subsequent 30-day chronic high temperature exposure (32 °C). Through integrated analyses of morphological parameters, texture characteristics, TUNEL assay, gene expression analysis, and metabolomics in muscle, the effects of high temperature stress on the meat quality of largemouth bass were systematically examined. The results showed that high temperature stress significantly upregulated key genes in the ubiquitin-proteasome pathway (*trim13*, *foxo1α*) and key genes in the autophagy-lysosome pathways (*lc3α*, *lc3β*, *bcl2l1*, *ctsl2*), induced apoptosis in muscle cells, and led to significant reductions in myofiber diameter and density. In terms of textural properties, high temperature stress significantly decreased parameters such as springiness, adhesiveness, and cohesiveness, as well as water holding capacity. Metabolomic analysis further revealed that high temperature induced remodeling of energy metabolism and significant reprogramming of purine and amino acid metabolic pathways, resulting in decreased levels of key flavor compounds, including IMP, GMP, flavor amino acids (glutamic acid, alanine, methionine, arginine, proline), and peptides (glu-glu-lys and glu-cys-gly), thereby adversely affecting muscle flavor quality. The findings of this study provide a theoretical basis for understanding the impact of thermal stress on the eating quality of farmed fish.

## Simple Summary

Although the negative effects of high temperature stress on fish growth and health have been extensively studied, its impact on muscle quality remains poorly understood. This is a study aimed to investigate how thermal stress affects the muscle quality of the largemouth bass *Micropterus salmoides* by integrating analyses of muscle morphology, texture, cell apoptosis, gene expression, and metabolomics. The results demonstrated that high temperature stress activates protein degradation pathways, induces muscle cell apoptosis, and reduces myofiber diameter and density, leading to deteriorated texture and decreased water-holding capacity. Furthermore, heat stress reprograms energy metabolism and alters purine and amino acid metabolic pathways, resulting in the depletion of key flavor compounds such as inosine monophosphate, flavor amino acids, and peptides. These findings provide new insights into the physiological and metabolic mechanisms by which thermal stress impairs fish muscle quality and offer a scientific basis for developing strategies to mitigate quality deterioration in farmed fish under high temperature conditions.

## 1. Introduction

In recent years, rising global temperatures have led to increasing water temperatures, posing a critical environmental challenge to the sustainable development of aquaculture [1,2,3]. It is estimated that thermal stress inflicts substantial economic losses on the global aquaculture industry [4]. These losses include direct mortality events as well as indirect production declines due to reduced growth rates, impaired disease resistance, and deteriorated muscle quality [5,6,7,8]. Temperature-sensitive species, such as the Yangtze sturgeon (*Acipenser dabryanus*), Nile tilapia (*Oreochromis niloticus*), and Largemouth bass (*Micropterus salmoides*) in intensive farming systems, are particularly vulnerable to thermal stress [9,10,11]. This not only limits production capacity but also threatens the stability of the aquaculture supply chain [12]. Consequently, thermal stress is increasingly recognized as a serious concern in aquaculture.

In aquaculture practice, thermal stress can be categorized based on duration and intensity into two primary types: acute thermal stress and rapid temperature increase followed by sustained thermal stress, which exert distinct influences on the physiological regulatory pathways and final quality of farmed fish [13]. Acute thermal stress typically refers to a short-term, rapid temperature increase, which primarily activates the neuroendocrine stress axis (e.g., the hypothalamic pituitary interrenal axis (HPI)), leading to the swift release of stress hormones such as cortisol and inducing the rapid expression of heat shock proteins to maintain cellular homeostasis [14,15]. This intense stress response often results directly in metabolic disturbances and energy depletion in fish, potentially accelerating glycolysis and lactate accumulation [16].

In contrast, chronic thermal stress arises from prolonged exposure to sublethal elevated water temperatures, with more insidious and cumulative effects. At the physiological level, a core impact of chronic thermal stress is the sustained activation of the neuroendocrine system [17]. The long-term excitation of the HPI leads to the maintenance of high levels of glucocorticoids, such as cortisol [18]. While beneficial for energy mobilization in the short term, chronically elevated cortisol can trigger excessive catabolism of proteins and lipids, suppress growth, and potentially lead to immunosuppression [19]. Furthermore, chronic thermal stress induces a comprehensive reprogramming of energy metabolism. To meet persistent energy demands, the organism’s basal metabolic rate typically increases [20]. This shift in energy allocation strategy diverts more resources towards maintaining homeostasis and mounting stress responses, rather than supporting growth and energy storage [21]. Although numerous studies have focused on the effects of thermal stress on physiological functions, tissue damage, and disease resistance in fish [22,23,24,25]. Systematic research on how thermal stress influences the muscle quality of economically important fish species remains relatively limited.

The largemouth bass is an economically important freshwater aquaculture species that exhibits marked sensitivity to thermal stress [26]. Its optimal growth temperature ranges from 25 to 28 °C, and sustained water temperatures exceeding 30 °C readily induce stress responses, including metabolic dysregulation, immune suppression, and growth retardation [27]. However, the effects of high temperature stress on the muscle quality of largemouth bass remain poorly understood. This study investigated the effects of different thermal stress conditions on the muscle quality of largemouth bass by simulating two typical thermal regimes: an acute temperature increase and an initial rapid temperature increase followed by sustained exposure. Histological staining and texture profile analysis were employed to quantitatively assess alterations in myofiber morphology and muscle textural properties. Combined with untargeted metabolomics, the response patterns of muscle metabolite profiles under high temperature stress and their potential regulatory mechanisms were further elucidated. The results aim to provide a theoretical basis for mitigating the adverse impacts of thermal stress on flesh quality in aquaculture.

## 2. Materials and Methods

### 2.1. Fish Feeding and High Temperature Exposure Trial

**Figure 1:**
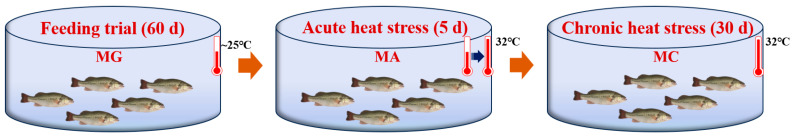
Schematic overview of the experimental design. Illustration of the sequential procedures involving growth experiment followed by acute and chronic thermal stress exposures in the largemouth bass *Micropterus salmoides*. MG: Feeding trial; MA: Acute heat stress; MC: Chronic heat stress.

Largemouth bass were obtained from a hatchery (Benao Agricultural Co., Ltd., Huzhou, China) and acclimatized in a 22 m^2^ fiberglass pool with an indoor recirculating aquaculture system. Prior to the formal experiment, the fish were fasted for 24 h and then anesthetized using tricaine mesylate (MS-222). A total of 132 fish with an average initial body weight of 45.73 ± 9.76 g, uniform size, and no superficial injuries were randomly selected and allocated into three replicate 1000 L fiberglass tanks, with 44 fish per tank. During the 60-day growth trial, fish were fed a diet to apparent satiation three times daily. The diet contained 55% crude protein, 10% crude lipid, and 8% moisture. At the end of this period, a subset of fish was sampled. Following the growth trial, the remaining fish were subjected to an acute thermal stress experiment. Water temperature was gradually and uniformly increased using aquarium heaters over 5 days until it reached 32 °C, at which point sampling was conducted. Subsequently, the remaining fish underwent a chronic thermal stress experiment, where they were maintained at a constant water temperature of 32 °C for 30 days before final sampling. The experimental procedure is shown in Figure 1. Throughout the entire culture period, water quality was maintained within the following ranges: dissolved oxygen > 6 mg/L, total ammonia-nitrogen < 0.1 mg/L, under a 12 h light:12 h dark photoperiod. Water temperature was continuously monitored. Feeding was continued, and mortality was recorded daily during both the acute and chronic high temperature exposures.

### 2.2. Sample Collection

At the end of the growth trial, 8 fish from each tank were randomly selected and anesthetized with MS-222. The dorsal muscle was exposed by dissection along the lateral line. Using sterile surgical instruments, a sample of dorsal muscle was excised from the left side of the body and divided into three portions. The first portion (approximately 0.5 cm^3^) was immediately fixed in 4% paraformaldehyde solution at 4 °C for 24 h for subsequent paraffin embedding, hematoxylin-eosin (H&E) staining, and TUNEL staining analysis. The second portion was placed in RNA protection solution and stored at −80 °C for gene expression analysis. The third portion (approximately 0.5 g) was flash frozen in liquid nitrogen and stored at −80 °C for later metabolomic analysis. Samples collected for gene expression and metabolomics analyses from every 4 fish from each tank were pooled and placed into a single cryotube. This procedure resulted in 2 cryotubes per tank. All procedures were performed on ice, and samples were carefully handled to avoid repeated freeze–thaw cycles. From the corresponding region on the right side of the body, the dorsal muscle was collected and trimmed with a surgical blade into a regular cube (approximately 1.0 cm × 1.0 cm × 1.0 cm), and a regular cubic muscle sample (approximately 1.0 cm × 1.0 cm × 0.5 cm) was excised from the abdominal region in the fish. Care was taken to ensure that the cut surfaces were smooth, and the orientation of the muscle fibers was aligned with the direction of subsequent compression. The prepared sample was then used for texture parameter determination using a texture analyzer (TMS-Pilot, FTC, Atlanta, GA, USA). The sampling sites and measured parameters in this experiment are presented in Table 1. Samples collected after the growth experiment, acute heat stress, and chronic heat stress were designated as the MG, MA, and MC groups, respectively.

### 2.3. Histological Examination

H&E staining of dorsal muscle tissues was carried out by Servicebio Company (Wuhan, China). The images of samples from each slide were obtained by an optical microscope (CX33RTFS2C, Olympus, Tokyo, Japan). For each muscle section per fish, three random fields of view were selected for morphological analysis. Quantitative analysis was performed using Image J (1.8.0) software to determine myofiber diameter and density (*n* = 6). Myofiber diameter was expressed as the Feret diameter, defined as the maximum distance between two parallel lines tangent to the contour of the fiber. Myofiber density was calculated as the ratio of the total cross-sectional area of muscle fibers within a field to the total area of that field. All measurements were conducted on images captured under consistent magnification.

### 2.4. TUNEL Assay

Dorsal muscle samples were fixed in 4% paraformaldehyde solution for over 24 h, followed by dehydration and embedding in paraffin blocks. Apoptotic cells in muscle sections from both the MG, MA, and MC groups were detected using the Terminal deoxynucleotidyl transferase-mediated dUTP Nick-End Labeling (TUNEL) assay. Muscle sections were subjected to TUNEL staining and counterstained with DAPI (4′,6-diamidino-2-phenylindole) according to the method described by Li et al. [28]. For quantification, six random fields per section were imaged at 100× magnification using a fluorescence microscope. The percentage of TUNEL-positive apoptotic cells was determined in these fields. All images were quantitatively analyzed using Image-Pro Plus (7.0) software.

### 2.5. Gene Expression Analysis

Total RNA was isolated from dorsal muscle tissues using Trizol Reagent (Vazyme, Nanjing, China), following the methodology described in a previous study [29]. RNA concentration and integrity were assessed with a Nanodrop 2000 spectrophotometer (Thermo, Waltham, MA, USA) and 1% agarose gel electrophoresis (Bio-Rad, Hercules, CA, USA), respectively. Complementary DNA (cDNA) was synthesized from the extracted RNA using a reverse transcription kit (Vazyme, Nanjing, China) in accordance with the manufacturer’s instructions. Quantitative real-time PCR (qPCR) was performed on an ABI 7500 real-time PCR system (Applied Biosystems, Carlsbad, CA, USA) in a 20 μL reaction volume. The thermal cycling conditions consisted of an initial step at 95 °C for 30 s, followed by 40 cycles of denaturation at 95 °C for 5 s and annealing/extension at 60 °C for 30 s. Following every run, a melting curve analysis was carried out to confirm the presence of only one amplification product. Prior to the experiment, the stability of β-actin in largemouth bass was evaluated. After confirming its stability, it was used as a reference gene for normalization. This internal reference gene was referenced from Gong et al. [30]. Gene expression levels were calculated using the 2^−ΔΔCt^ method [31]. All primer sequences used in qPCR are listed in Table 2.

### 2.6. Texture Profile Analysis

Texture profile analysis (TPA) of dorsal and abdominal muscle samples was performed using a texture analyzer. A 75 mm cylindrical compression probe (TMS75mm, FTC, Atlanta, GA, USA) was used to compress the samples at a speed of 1 mm/s to 30% of their original height, then retracted. After a 5 s interval, a second compression was applied under the same conditions. From the resulting TPA curves, the following key parameters were extracted and calculated: springiness, hardness, gumminess, adhesiveness, cohesiveness, and chewiness. Water holding capacity, which reflects the ability of muscle to retain water, was evaluated by measuring centrifugal water loss and cooking loss according to the method described by Lv et al. [32].

### 2.7. Untargeted UPLC-TOF/MS Metabolomic Analysis

Dorsal muscle samples from the MG group and MC group were thawed on ice and homogenized in liquid nitrogen using a ball mill (MU-G02-0448, Miou Instrument Co., Hangzhou, China). Approximately 20 mg of homogenized tissue was weighed into a 2 mL tube and extracted with 400 μL of 70% methanol containing internal standards. After vortexing (1500 rpm, 5 min) and incubation on ice (15 min), the mixture was centrifuged (12,000 rpm, 10 min, 4 °C). A 300 μL aliquot of supernatant was transferred to a new tube, held at −20 °C for 30 min, and centrifuged again (12,000 rpm, 3 min, 4 °C). Finally, 200 μL of supernatant was collected for LC-MS analysis. Metabolite separation was performed on a Vanquish UPLC system (Thermo Scientific, Waltham, MA, USA) equipped with an ACQUITY Premier HSS T3 column (1.8 μm, 2.1 × 100 mm; Waters, Milford, MA, USA) maintained at 40 °C. The mobile phase consisted of 0.1% formic acid in water (A) and 0.1% formic acid in acetonitrile (B) at a flow rate of 0.4 mL/min, with an injection volume of 4 μL. Metabolites were detected using a Q Exactive HF-X mass spectrometer (Thermo Scientific, Waltham, MA, USA) in both full-scan and MS/MS modes. The electrospray ionization source was operated at 3.5 kV, with sheath gas at 30 arb, auxiliary gas at 5 arb, capillary temperature of 320 °C, and vaporizer temperature of 300 °C. All data were processed and normalized using MetaboAnalyst 5.0 software for analyzing differences in metabolite levels in the dorsal muscle of largemouth bass between the growth group and the heat-stress group. Metabolites meeting the thresholds of |log_2_(fold change)| > 2 and an adjusted *p* value (Q value) ≤ 0.05 were identified as significantly differential metabolites.

### 2.8. Determination of Flavor Nucleotide Concentrations

Flavor nucleotide contents (Inosine monophosphate (IMP), guanosine monophosphate (GMP), and adenosine monophosphate (AMP) in flesh were determined by high-performance liquid chromatography (HPLC). Briefly, flesh samples (5 g) were homogenized in 10 mL of 0.1 M perchloric acid at 4 °C for 15 min, then centrifuged at 1840× *g* for 10 min. The pellet was re-extracted with 5 mL of perchloric acid. The combined supernatants were neutralized to pH 6.4 with 1 M KOH, adjusted to 50 mL, and filtered (0.45 μm). A 20 μL aliquot was injected into an HPLC system (Waters 2695e, USA) equipped with a C18 column (4.6 × 250 mm, 5 μm). Mobile phase A: 0.01 M KH_2_PO_4_; mobile phase B: methanol. Column temperature: 30 °C; flow rate: 1.0 mL/min; UV detection: 254 nm.

### 2.9. Statistical Analysis

All statistical analyses were performed using SPSS 20.0. Data are presented as mean ± standard deviation, and graphs were generated with Origin 2022 (9.9) software. Statistical analyses were performed using one-way analysis of variance (ANOVA), followed by Duncan’s multiple range test for post hoc comparisons between treatment groups. Prior to these tests, the normality of data distribution was verified with the Kolmogorov–Smirnov test, and homogeneity of variances was assessed using Levene’s test. Differences were considered statistically significant at *p* < 0.05. An independent samples Student’s *t*-test was conducted to analyze whether there was a significant difference in the expression levels of key genes and the relative abundance of flavor metabolites between the MG and MC groups. Statistical significance is denoted by asterisks: *p* < 0.05 (*), *p* < 0.01 (**), and *p* < 0.001 (***).

## 3. Results

### 3.1. Effects of Different Thermal Stress Regimes on Morphological Characteristics of Myofiber in Largemouth Bass Micropterus salmoides

**Figure 2:**
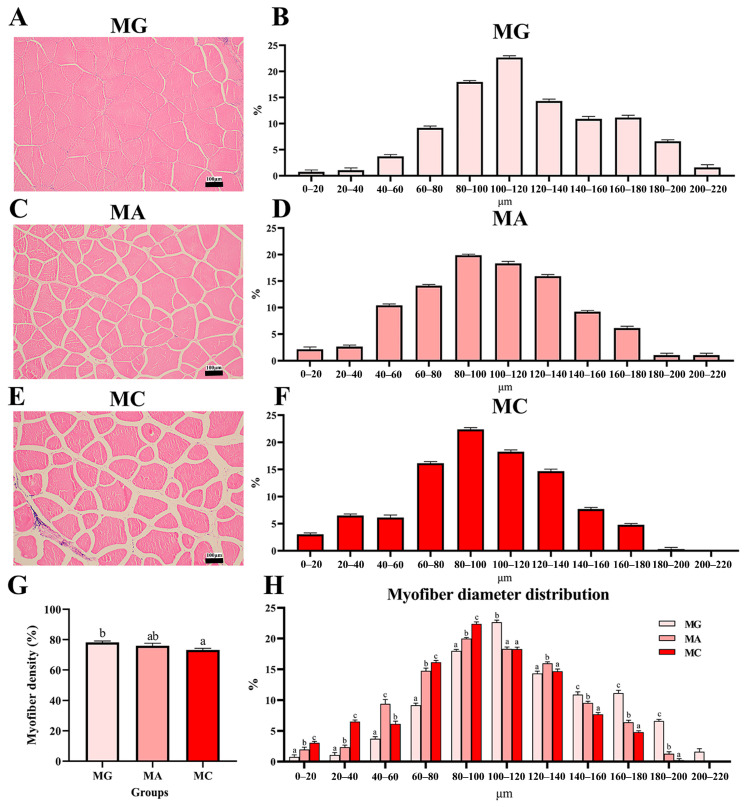
Effects of different thermal stress regimes on myofiber diameter and density in largemouth bass. (**A**,**C**,**E**) Histological characteristics of myofiber in largemouth bass under different thermal stress conditions. (**B**,**D**,**F**) Histograms of myofiber diameter of largemouth bass under different thermal stress conditions. (**G**) Quantitative graph of H &E staining, percentage of myofiber area of the section area. (**H**) Distribution frequency of Myofiber diameters in largemouth bass under different thermal stress conditions. MG: Feeding trial; MA: Acute heat stress; MC: Chronic heat stress. Results are shown as means ± SEM (*n* = 6). For each index, bars without sharing a common letter indicate significant differences (*p* < 0.05).

The effects of different thermal stress regimes on morphological parameters of myofibers are shown in Figure 2. H&E-stained sections of dorsal muscle revealed increased inter-fibrillar spacing following acute heat stress, which became more pronounced after subsequent chronic heat stress (Figure 2A–F). The quantitative results of myofiber density indicated a decrease in the MA group and a significant reduction in the MC group relative to the MG group (*p* < 0.05) (Figure 2G). Frequency distribution analysis of myofiber diameters showed that the MA group exhibited significantly higher frequencies in the diameter ranges of 0–20 μm, 20–40 μm, 40–60 μm, 60–80 μm, and 80–100 μm compared to the MG group (*p* < 0.05). The MC group displayed significantly higher frequencies in the 0–20 μm, 20–40 μm, 60–80 μm, and 80–100 μm ranges relative to both the MG and MA groups (*p* < 0.05). Conversely, the frequencies of diameters in the ranges of 100–120 μm, 140–160 μm, 160–180 μm, and 180–200 μm were significantly lower in the MA group than in the MG group, and similarly lower in the MC group compared to the MG group (*p* < 0.05). Moreover, the MC group showed significantly lower frequencies in the 140–160 μm, 160–180 μm, and 180–200 μm ranges relative to the MA group (*p* < 0.05). Notably, no myofibers exceeding 200 μm in diameter were observed in either the MA or MC groups (Figure 2H).

### 3.2. Effects of Different Thermal Stress Regimes on Apoptosis and Gene Expression Related to Protein Metabolism in Largemouth Bass Micropterus salmoides

**Figure 3:**
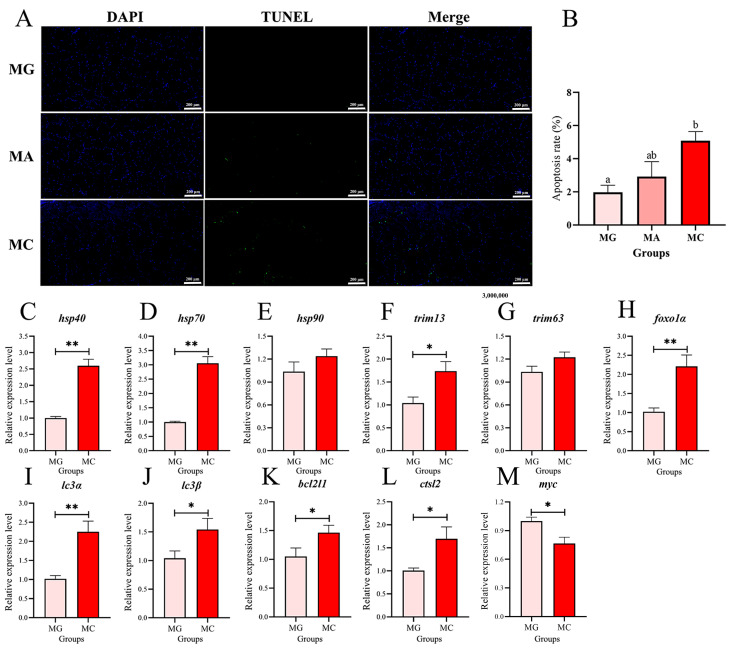
Effects of thermal stress on apoptosis and gene expression related to protein degradation in the muscle of the largemouth bass. (**A**) TUNEL staining, under fluorescence microscope, apoptotic cells appeared green fluorescence, while normal nuclei appeared blue color. Photomicrographs magnification (100×) and scale bar (200 μm). (**B**) Apoptosis rate (%). Columns represent the mean ± SEM (*n* = 6). For each index, bars without sharing a common letter indicate significant differences (*p* < 0.05). (**C**–**M**) Relative expression levels of *hsp40*, *hsp70*, *hsp90*, *trim13*, *trim63*, *foxo1α*, *lc3α*, *lc3β*, *bcl2l1*, *ctsl2*, *myc*. MG: Feeding trial; MA: Acute heat stress; MC: Chronic heat stress. For each index, “*” indicated that *p* < 0.05 between two groups, “**” indicated that *p* < 0.01 between two groups (*n* = 6).

Muscular apoptosis in largemouth bass was assessed via TUNEL assay with DAPI counterstaining, and gene expression related to protein degradation is presented in Figure 3. Fluorescence microscopy revealed significant apoptosis signals in fish after thermal exposure (Figure 3A). Quantitative analysis further showed that the apoptosis rate in the MA group was higher than that in the MG group, whilst the apoptotic rate in the MC group was significantly larger than that in the MG group (*p* < 0.05) (Figure 3B). To further investigate the molecular mechanisms underlying the effects of high-temperature thermal stress on largemouth bass, we measured the expression levels of genes related to heat stress and protein metabolism in the MG and MC groups. The expression levels of key heat shock protein genes are shown in Figure 3C–E. Compared to the MG group, the expression of *hsp40* and *hsp70* was significantly up-regulated in the MC group (*p* < 0.05) (Figure 3C,D). The expression levels of key genes involved in protein degradation pathways, namely the ubiquitin-proteasome pathway and the autophagy-lysosome pathway, are presented in Figure 3F–L. Following thermal stress, the expression of *trim13* and *foxo1α*, genes associated with the ubiquitin-proteasome pathway, also increased significantly (*p* < 0.05) (Figure 3F,H). In the autophagy-lysosome pathway, the expression of key genes *lc3α*, *lc3β*, *bcl2l1*, and *ctsl2* was significantly higher in the MC group than in the MG group (*p* < 0.05) (Figure 3I–L). In addition, *myc* expression in the MC group was significantly lower than in the MG group (*p* < 0.05) (Figure 3M). Although the expression levels of *hsp90* and *trim63* were elevated in the MC group compared to the MG group, the differences were not statistically significant (Figure 3E,G).

### 3.3. Effects of Different Thermal Stress Regimes on Textural Properties of Dorsal Muscle in Largemouth Bass Micropterus salmoides

**Figure 4:**
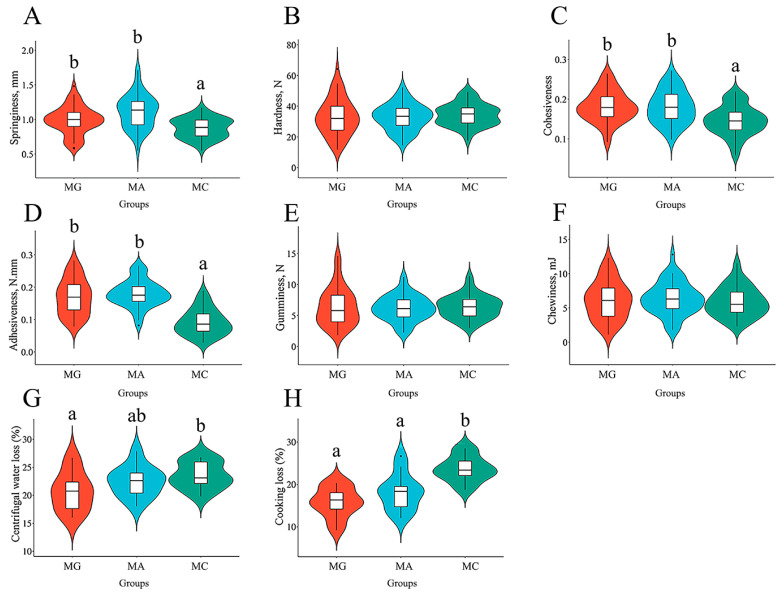
Effects of different thermal stress regimes on textural properties of dorsal muscle in largemouth bass. Violin diagram showing the textural characteristics of largemouth bass. (**A**–**H**) Springiness; Hardness; Cohesiveness; Adhesiveness; Gumminess; Chewiness; Centrifugal water loss; Cooking loss. MG: Feeding trial; MA: Acute heat stress; MC: Chronic heat stress. For each index, bars without sharing a common letter indicate significant differences. Significant differences were determined at *p* < 0.05.

The effects of different thermal stress regimes on the textural properties of dorsal muscle are shown in Figure 4. In the dorsal muscle, springiness was significantly lower in the MC group compared to both the MA and MG groups (*p* < 0.05) (Figure 4A). Cohesiveness and adhesiveness were significantly lower in the MC group than in the MA and MG groups (*p* < 0.05) (Figure 4C,D). Meanwhile, centrifugal water loss in the dorsal muscle of the MC group was significantly higher than that in the MG group (*p* < 0.05) (Figure 4G). Regarding cooking loss, the MC group showed significantly higher values than the MG and MA groups (*p* < 0.05) (Figure 4H). The aforementioned results indicate that thermal stress significantly reduces the water-holding capacity of the dorsal muscle in largemouth bass. No significant differences were observed in hardness, gumminess, or chewiness among the three treatment groups (Figure 4B,E,F).

### 3.4. Effects of Different Thermal Stress Regimes on Textural Properties of Abdominal Muscle in Largemouth Bass Micropterus salmoides

**Figure 5:**
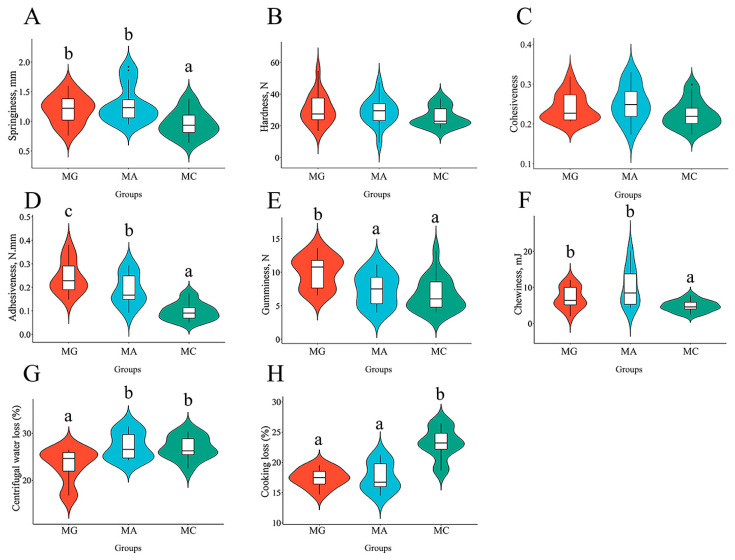
Effects of different thermal stress regimes on textural properties of abdominal muscle in largemouth bass. Violin diagram showing the textural characteristics of largemouth bass. (**A**–**H**) Springiness; Hardness; Cohesiveness; Adhesiveness; Gumminess; Chewiness; Centrifugal water loss; Cooking loss. MG: Feeding trial; MA: Acute heat stress; MC: Chronic heat stress. For each index, bars without sharing a common letter indicate significant differences. Significant differences were determined at *p* < 0.05.

The effects of different thermal stress regimes on textural properties of abdominal muscle are shown in Figure 5. In the abdominal muscle, springiness was significantly lower in the MC group compared to both the MG and MA groups (*p* < 0.05) (Figure 5A). Adhesiveness was significantly higher in the MG group than in the MA group, whereas the MC group exhibited significantly lower adhesiveness than both the MG and MA groups (*p* < 0.05) (Figure 5D). Gumminess was significantly greater in the MG group relative to the MA and MC groups (*p* < 0.05) (Figure 5E). Chewiness was significantly reduced in the MC group compared to the MG and MA groups (*p* < 0.05) (Figure 5F). Regarding water holding capacity, both the MA and MC groups showed significantly higher centrifugal loss than the MG group (*p* < 0.05) (Figure 5G). Cooking loss was significantly increased in the MC group compared to the MG and MA groups (*p* < 0.05) (Figure 5H). These results indicate that thermal stress significantly impairs the water-holding capacity of abdominal muscle in largemouth bass.

### 3.5. Effects of Thermal Stress on the Metabolomic Features in Largemouth Bass Micropterus Salmoides

**Figure 6:**
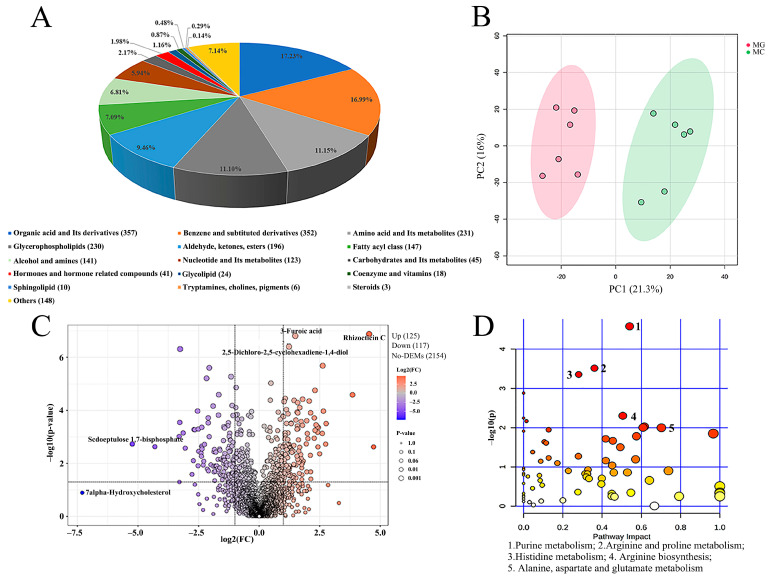
Effects of high temperature stress on metabolites of dorsal muscle in largemouth bass. (**A**) The pie chart exhibits the biochemical categories of the metabolites identified in muscle. (**B**) Principal component analysis (PCA) score plots for metabolomics of dorsal muscle (MC vs. MG). (**C**) The volcano plot illustrates the variation in metabolites of largemouth bass after a growth trial and chronic thermal stress. The orange dots represent up-regulated metabolites. The purple dots represent significantly down-regulated metabolites. (**D**) KEGG pathway enrichment analysis of pathway enrichment in MC vs. MG. MG: Feeding trial; MC: Chronic heat stress. The color of the dot indicates significance, the redder the dot, the more significant. The size of the dots represents the number of enriched metabolites.

To gain a deeper understanding of the effects of high-temperature thermal stress on muscle metabolites in largemouth bass, metabolomics analysis was performed on dorsal muscle samples from the MG and MC groups using liquid chromatography-mass spectrometry. A total of 2072 metabolites were identified, primarily categorized as follows: 357 organic acids and derivatives (17.23%), 352 benzene and substituted derivatives (16.99%), 231 amino acids and their metabolites (11.15%), 230 glycerophospholipids (11.10%), and 196 aldehydes, ketones, and esters (9.46%) (Figure 6A). Principal component analysis (PCA) score plot revealed a clear separation between the metabolic profiles of the MC and MG groups, indicating significant differences in metabolite composition (Figure 6B). Volcano plot analysis showed that the relative abundance of 125 metabolites significantly increased, while that of 117 metabolites significantly decreased after thermal stress (Figure 6C). Kyoto Encyclopedia of Genes and Genomes (KEGG) pathway enrichment analysis of the identified metabolites highlighted significantly enriched pathways, as illustrated in the bubble plot (Figure 6D) and Table 3. Among these, purine metabolism exhibited the highest −log(*p*) value and was identified as the most markedly affected pathway in muscle, followed by arginine and proline metabolism, histidine metabolism, arginine biosynthesis, and alanine, aspartate, and glutamate metabolism.

### 3.6. Effects of Thermal Stress on Taste Metabolites of Muscle in Largemouth Bass Micropterus salmoides

**Figure 7:**
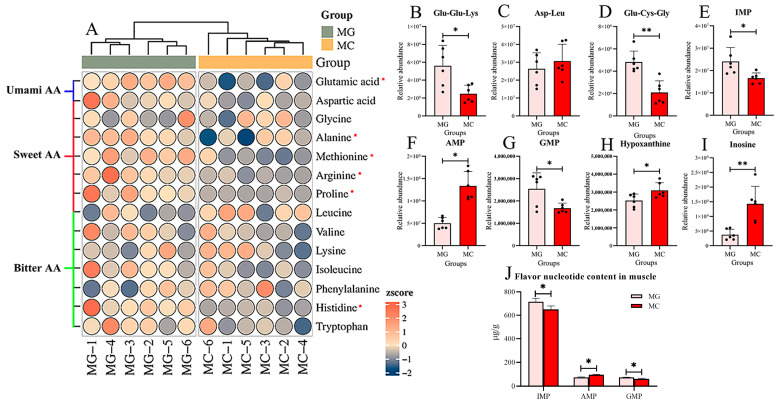
Effects of high temperature stress on taste metabolites of dorsal muscle in largemouth bass. (**A**) Heatmap of taste amino acids relative abundance in largemouth bass from MG and MC Groups. Metabolites marked with an asterisk “*” indicate significant differences in relative abundance. Umami amino acids (AA): Glutamic acid, Aspartic acid. Sweet amino acids: Aspartic acid, Glycine, Alanine, Methionine, Arginine, Proline, Leucine. Bitter amino acids: Leucine, Valine, Lysine, Isoleucine, Phenylalanine, Histidine, Tryptophan. (**B**–**D**) Relative abundance of flavor peptides: Glu-Glu-Lys, Asp-Leu, Glu-Cys-Gly. (**E**–**I**) Relative abundance of flavor nucleotides: IMP, AMP, GMP, (**J**) The contents of IMP, AMP, and GMP in muscle. hypoxanthine, inosine. MG: Feeding trial; MC: Chronic heat stress. Columns represent the mean ± SEM (*n* = 6). For each index, “*” indicated that *p* < 0.05 between two groups, “**” indicated that *p* < 0.01 between two groups.

Heatmap analysis illustrates the changes in taste-active amino acids in the muscle of the largemouth bass following thermal stress. The levels of the umami-associated amino acid glutamic acid, the sweet-tasting amino acids alanine, methionine, arginine, and proline, and the bitter-tasting amino acid histidine were significantly decreased (*p* < 0.05) (Figure 7A). The relative abundances of two taste-active peptides, Glu-Glu-Lys and Glu-Cys-Gly, were also significantly reduced (*p* < 0.05) (Figure 7B,D). Regarding flavor nucleotides, compared with the MG group, the relative abundances of IMP and GMP in the MA group were significantly decreased, whereas those of AMP, hypoxanthine, and inosine were significantly increased (*p* < 0.05) (Figure 7E–I). The contents of flavor nucleotide content in muscle tissues were determined by HPLC and are shown in Figure 7J. This result is similar to that of metabolomics; the contents of IMP and GMP in the MC group were significantly lower than those in the MG group (*p* < 0.05), while the AMP content in the MC group was significantly lower than that in the MG group (*p* < 0.05).

## 4. Discussion

### 4.1. High Temperature Stress Alters Muscle Fiber Morphology and Causes Deterioration in Muscle Texture

Textural properties are key indicators for evaluating fish quality, primarily including springiness, hardness, cohesiveness, adhesiveness, chewiness, and gumminess [33]. In addition to genetic factors, environmental conditions also serve as significant variables influencing the formation of muscle texture in fish [34]. This study further confirms that high temperature stress also significantly alters the textural properties of fish muscle. Texture profile analysis of dorsal and abdominal muscles revealed that high temperature stress reduced the springiness, cohesiveness, and adhesiveness of largemouth bass muscle, thereby contributing to an overall decline in muscle quality. Currently, research on the effect of muscle texture due to high temperature stress has been more commonly reported in poultry, whereas related studies in fish remain relatively limited [35,36]. Furthermore, this study found that compared to acute high temperature stress alone, the combined stress regime of acute warming followed by sustained high temperature exerted a more pronounced effect on the textural parameters of largemouth bass muscle. Muscle water holding capacity (WHC) is closely associated with meat tenderness, juiciness, and flavor [37]. Muscles with high WHC exhibit less water loss during cooking, resulting in a more tender and juicy texture, along with better retention of flavor compounds. In contrast, poor WHC often leads to dry and tough flesh, negatively affecting eating quality. Cooking loss and centrifugal water loss are commonly used indicators for evaluating WHC [32]. The results of this study revealed that high temperature stress significantly increased both cooking loss and centrifugal loss in largemouth bass muscle, indicating a marked decline in WHC. Similar phenomena have also been reported in studies on tilapia and rainbow trout (*Oncorhynchus mykiss*) [38,39]. Furthermore, this study demonstrated that sustained high temperature stress following acute heat exposure also significantly impaired the WHC of largemouth bass muscle.

As the basic structural unit of muscle tissue, the morphological characteristics of myofibers directly influence textural attributes such as tenderness, hardness, and chewiness [40]. The present study found that acute high temperature stress led to reductions in myofiber diameter and density, and these changes were further exacerbated under the stress regime of acute warming followed by sustained high temperature. Furthermore, TUNEL assay results indicated that high temperature stress induced apoptosis in muscle cells. The autophagy-lysosomal pathway and the ubiquitin-proteasome pathway are two major routes for intracellular protein degradation [41]. Gene expression analysis in this study revealed that both pathways were significantly activated under thermal stress, suggesting that muscle protein metabolism in largemouth bass shifted toward an enhanced catabolic state during high temperature exposure. Collectively, these findings suggest that high temperature likely impairs the structural integrity of myofibers and contributes to the reduction in myofiber density by concurrently activating protein degradation pathways and promoting cellular apoptosis. Myofiber diameter and density are highly correlated with muscle WHC and related indicators [42]. Zhang et al. [43] noted that dietary aflatoxin B1 supplementation reduced the elasticity and stickiness of yellow catfish muscle by decreasing myofiber diameter and density (*Pelteobagrus fulvidraco*). Another study on gibel carp (*Carassius auratus gibelio*) reported that increased water flow velocity enhanced myofiber diameter and density, thereby improving muscle WHC [44]. The findings of this study further elucidate that high temperature stress may impair muscle WHC and textural properties such as springiness by altering myofiber morphology, ultimately leading to a decline in the muscle quality of largemouth bass.

### 4.2. Metabolomics Reveals That High Temperature Stress Induces Significant Alterations in Purine Metabolism and Amino Acid-Related Metabolic Pathways

Metabolomics serves as a key technique for systematically analyzing dynamic metabolic responses in animals under environmental stress [45,46]. In this study, PCA revealed clear separation between MG and MC groups, indicating significant alterations in the muscle metabolic profile of largemouth bass following thermal stress. Enrichment analysis of differential metabolites provided a comprehensive overview of metabolic changes before and after high temperature exposure in the cultured species [47,48]. The present study showed that the purine metabolism pathway and the amino acid-related metabolic pathways were the most significantly affected. From a physiological perspective, under high temperature stress, animal organisms experience an elevated metabolic load, necessitating the mobilization of substantial energy to sustain essential life functions and counteract potential oxidative damage and inflammatory responses induced by thermal exposure [49,50]. ATP, a crucial molecule in energy metabolism and a central intermediate in purine metabolism, reflects the state of energetic demand [51]. Meanwhile, amino acids, as primary products of protein catabolism for energy supply, further corroborate the elevated energy requirements under thermal stress. In this study, the significant enrichment of purine and amino acid metabolism pathways reveals, at the metabolic level, that the physiological adaptation strategy of largemouth bass in response to prolonged high temperature stress centers on the reconfiguration of energy metabolism and the adjustment of nitrogen metabolism. Similarly, Ouyang et al. [52] reported significant enrichment of amino acid-related pathways in the thermal stress response of the clam (*Cyclina sinensis*), while Jiang et al. [53] using oxygen consumption and ammonia excretion rates, demonstrated that amino acids contribute to oxidative energy production via deamination during high temperature stress. Notably, both purine and amino acid metabolism pathways encompass various metabolites associated with flavor formation. In addition, the depletion of flavor-related metabolites in the arginine and proline metabolism pathway may be attributed to the downregulation of key enzymes such as arginase and proline dehydrogenase under chronic thermal stress, which limits the conversion of arginine to ornithine and proline to glutamate [54,55]. This enzymatic shift likely reduces the availability of precursor metabolites for nucleotide synthesis, thereby impairing the accumulation of IMP and other flavor compounds. Further analysis of changes in these metabolites is warranted to elucidate the mechanisms through which high temperature stress affects the flavor quality of largemouth bass muscle.

### 4.3. High Temperature Stress Causes a Significant Reduction in Muscle Taste Metabolites, Thereby Impairing Muscle Flavor Quality

Taste is a key determinant of the eating quality of meat [56]. Both the purine metabolism pathway (e.g., IMP, GMP) and amino acid metabolism pathways (e.g., glutamic acid, arginine, glycine) contain a variety of taste-active compounds closely associated with the formation of umami and sweet flavors [57]. Alterations in the levels of these metabolites directly influence the final flavor profile of muscle. In this study, it was found that after exposure to high temperature stress, the relative abundance of umami amino acid glutamic acid, as well as sweet taste amino acids including alanine, methionine, arginine, and proline, were significantly reduced in the muscle of largemouth bass. This alteration is likely associated with the increased energy demand during thermal stress, as these amino acids may be mobilized for oxidative energy production to cope with metabolic pressure [58]. Similar phenomena have been observed in tilapia, in which glutamic acid content in dorsal muscle significantly decreased under high temperature stress, and comparable findings have also been reported in shellfish studies [38,53]. Furthermore, umami peptides constitute an important component of muscle taste [59]. This study, for the first time, revealed that high temperature stress significantly reduced the levels of umami peptides Glu-Glu-Lys and Glu-Cys-Gly in largemouth bass. This change may likewise be related to the redistribution of energy metabolism, though the precise mechanisms warrant further investigation.

IMP, AMP, and GMP are key flavor compounds that significantly influence the taste profile of fish muscle, with their contents showing a strong positive correlation with the perception of umami [60,61]. The results of this study indicate that after high temperature stress, the levels of IMP and GMP in the muscle of the largemouth bass were significantly reduced, while AMP levels increased markedly. IMP, AMP, and GMP are all purine nucleotides that can be interconverted within the metabolic network [62,63]. The observed changes are likely closely related to the remodeling of energy metabolism in fish under thermal stress. AMP serves as a key indicator of cellular energy status, and its pronounced elevation typically reflects an energy-stressed state in which rapid ATP consumption leads to the accumulation of its degradation product, AMP [64]. Concurrently, the decline in IMP and GMP content may be attributed, on one hand, to feedback inhibition of their synthesis pathways due to AMP accumulation, and on the other hand, to the possible channeling of IMP into the salvage pathway for rapid ATP generation under energy-deficient conditions, resulting in a net decrease in its concentration [65,66]. A significant reduction in muscle IMP content due to thermal stress has also been reported in studies on rainbow trout [39]. The interconnected conversion network among purine nucleotides undergoes dynamic adjustments in response to energy stress [67]. The metabolite alteration pattern observed in this study directly reflects the reprogramming of purine metabolism in largemouth bass as an adaptive strategy to maintain energy homeostasis under high temperature stress. Although such metabolic redirection may support short-term energy supply, it could also adversely affect the final flavor quality of the muscle due to the depletion of key taste-active compounds.

## 5. Conclusions

This study investigated the effects of high temperature stress on the muscle quality of largemouth bass by simulating two thermal regimes: acute warming and rapid warming followed by sustained high temperature. The results indicated that thermal stress activated both the ubiquitin-proteasome and autophagy-lysosomal protein degradation pathways, induced apoptosis in muscle cells, and led to significant reductions in myofiber diameter and density. In terms of texture, thermal stress significantly decreased parameters such as springiness and cohesiveness, along with water holding capacity. Acute temperature elevation markedly altered the morphological characteristics of fish muscle and promoted textural deterioration, while subsequent sustained high temperature further exacerbated these effects. Additionally, this study revealed the underlying metabolic mechanism through which thermal stress affects muscle flavor quality. High temperature induced a restructuring of energy metabolism, leading to significant reprogramming of purine and amino acid metabolic pathways. This metabolic shift resulted in the depletion of key flavor compounds, such as IMP, GMP, various umami-related amino acids, and peptides, thereby adversely affecting the overall flavor profile of the muscle. This study demonstrates that thermal stress can reduce muscle quality in largemouth bass by altering myofiber morphology, impairing textural properties, and lowering the levels of flavor-related metabolites. These findings provide a theoretical basis for understanding the physiological mechanisms by which elevated temperature affects muscle quality in fish.

## Figures and Tables

**Table 1:** Summary of sample collection details.

Period	Growth Trial	Acute Heat Stress	Chronic Heat Stress
Sampling location	Dorsal muscle	Dorsal muscle	Dorsal muscle
Measured parameters	H&E staining, TUNEL staining, Texture profile analysis, Gene expression analysis, Metabolomic analysis	H&E staining, TUNEL staining, Texture profile analysis	H&E staining, TUNEL staining, Texture profile analysis, Gene expression analysis, Metabolomic analysis

**Table 2:** Primer sequences for qRT-PCR analysis of mRNA expression.

Gene	Sense and Antisense Primer (5′-3′)	Size (bp)	Accession No.
*hsp40* ^1^	F: CTGCAGAGGGCTAAGGGAT R: GTCAGTGCAACCTCAGTCTC	82	XM_038714027.1
*hsp70* ^2^	F: AGCATCAGCAGAAGGAGCTG R: TAGTCGACCTCCTCGATGGT	175	XM_038693533.1
*hsp90* ^3^	F: GTCAGTTTGGCGTGGGTTTC R: GTCACGCTCCTTCTCCACAA	287	XM_038726807.1
*trim13* ^4^	F: TCTCAGCAGCTGGACACCAT R: TGTTCTGAAAGCCGGACCTC	162	XM_038720880.1
*trim63* ^5^	F: GACCAGCTGATCTGCCTGTAATC R: AGCAACAGACTGGGGATATGTG	189	XM_038719630.1
*lc3α* ^6^	F: AGGCCGTTGTACTGCACATT R: ATCACGGGGATCTTGTTGGG	156	XM_038698418.1
*lc3β* ^7^	F: TCTGCGAGGTGTATGAACGG R: GCAAGAAACATGGAGGACGC	171	XM_038705174.1
*foxo1α* ^8^	F: CTCAGCAGCTGGACACCAT R: CGGTTCCCTTCCAAGAGTCC	248	XM_038693765.1
*ctsl2* ^9^	F: GTGTTCTTCGCTCTTAGGGAG R: CCTCTCTGGTTTGTGCGTGTA	374	XM_038704446.1
*bcl2l1* ^10^	F: CTCATCCTGGCAGTTTGTCGT R: CTCCAGACAACCATAGCCCA	171	XM_022769814.1
*myh* ^11^	F: TCCCTACAAGTGGCTTCCTG R: TACTGGATGACACGCTTGGT	213	XM_038709909.1
*β-actin*	F: TTCACCACCACAGCCGAAAG R: TCTGGGCAACGGAACCTCT	179	KJ669298.1

Note: ^1^ *hsp40*, Heat shock protein 40; ^2^ *hsp70*, Heat shock protein 70; ^3^ *hsp90*, Heat shock protein 90; ^4^ *trim13*, Tripartite motif-containing 13; ^5^ *trim63*, Tripartite motif-containing 63; ^6^ *lc3α*, Microtubule-associated protein 1 light chain 3*α*; ^7^ *lc3β*, Microtubule-associated protein 1 light chain 3*β*; ^8^ *foxo1α*, Forkhead box protein O1*α*; ^9^ *ctsl2*, Cathepsin L2; ^10^ *bcl2l1*, B-cell lymphoma/leukemia-like gene 1; ^11^ *myh*, myosin heavy chain.

**Table 3:** KEGG pathway enrichment analysis of the metabolites at MC vs. MG (Top 20).

Pathway Name	*p*	−log(*p*)	FDR
Purine metabolism	2.64 × 10^−5^	4.579	0.0018455
Arginine and proline metabolism	3.04 × 10^−4^	3.5167	0.010258
Histidine metabolism	4.40 × 10^−4^	3.3569	0.010258
Valine, leucine, and isoleucine biosynthesis	0.0013148	2.8811	0.023009
Arginine biosynthesis	0.0049734	2.3033	0.066553
Nitrogen metabolism	0.0057046	2.2438	0.066553
Metabolism of xenobiotics by cytochrome P450	0.006799	2.1676	0.06799
Pyrimidine metabolism	0.0093713	2.0282	0.070296
Fructose and mannose metabolism	0.0099387	2.0027	0.070296
Alanine, aspartate, and glutamate metabolism	0.010042	1.9982	0.070296
Vitamin B6 metabolism	0.011301	1.9469	0.071545
Drug metabolism-cytochrome P450	0.012265	1.9113	0.071545
Nicotinate and nicotinamide metabolism	0.014122	1.8501	0.076041
beta-Alanine metabolism	0.016499	1.7825	0.082496
Steroid hormone biosynthesis	0.019308	1.7143	0.090104
Selenocompound metabolism	0.021601	1.6655	0.094419
Glycerolipid metabolism	0.02293	1.6396	0.094419
Valine, leucine, and isoleucine degradation	0.024487	1.6111	0.095226
Glycerophospholipid metabolism	0.031406	1.503	0.11571
Propanoate metabolism	0.041809	1.3787	0.14633

## Data Availability

The original contributions presented in the study are included in the article, and further inquiries can be directed to the corresponding authors.

## References

[1] Liu E., Zhao X., Li C., Wang Y., Li L., Zhu H., Ling Q. (2022). Effects of acute heat stress on liver damage, apoptosis and inflammation of pikeperch (*Sander lucioperca*). J. Therm. Biol..

[2] Mugwanya M., Dawood M.A.O., Kimera F., Sewilam H. (2022). Anthropogenic temperature fluctuations and their effect on aquaculture: A comprehensive review. Aquac. Fish..

[3] Mattos D.d.C., Cardoso L.D., Oliveira A.T., de Screnci-Ribeiro R., Mattos B.O., de Aride P.H.R., Radael M.C., Motta J.H.d.S., Vidal M.V. (2024). Effect of temperature on the embryonic and larvae development of discus fish *Symphysodon aequifasciatus* and time of first feeding. Zygote.

[4] Mei Y., Xu Y., Gao Q., Li Z., Dong S. (2025). Effects of temperature and salinity on CO_2_ fluxes dynamics and respiration metabolism in the sea cucumber *Apostichopus japonicus* (Selenka). Mar. Pollut. Bull..

[5] Fantini L.E., Smith M.A., Jones M., Roy L.A., Lochmann R., Kelly A.M. (2021). Growth parameters in northern largemouth bass *Micropterus salmoides salmoides* raised near their upper thermal tolerance for 28 days. Aquac. Res..

[6] Li Y., Zhou C., Zhang Y., Zhao X. (2024). Effects of heat stress on the muscle meat quality of rainbow trout. Fishes.

[7] Sun J., Zhao L., Wu H., Lian W., Cui C., Du Z., Luo W., Li M., Yang S. (2019). Analysis of miRNA-seq in the liver of common carp (*Cyprinus carpio* L.) in response to different environmental temperatures. Funct. Integr. Genom..

[8] Xv Z., Chen S., Song G., Hu H., Lin S., Long Y. (2024). Biochemical, histological and transcriptomic analyses for the immunological organs provide insights into heat stress-induced disease susceptibility in Largemouth Bass. Sci. Total. Environ..

[9] Chen Y., Wu X., Lai J., Liu Y., Song M., Li F., Gong Q. (2023). Integrated biochemical, transcriptomic and metabolomic analyses provide insight into heat stress response in Yangtze sturgeon (*Acipenser dabryanus*). Ecotoxicol. Environ. Saf..

[10] Qiang J., Tao Y.F., Zhu J.H., Lu S.Q., Cao Z.M., Ma J.L., He J., Xu P. (2022). Effects of heat stress on follicular development and atresia in Nile tilapia (*Oreochromis niloticus*) during one reproductive cycle and its potential regulation by autophagy and apoptosis. Aquaculture.

[11] Jian W., Gao J., Xia X., Cao W., Qin X., Lin H., Chen Z., Zheng H., Zhu G., Zheng Z. (2025). Environmental stressors in seafood supply chains: From molecular mechanisms to advanced preservation strategies. Food Chem..

[12] Tian M., Zhao S., Zhu X., Han D., Zhang L. (2026). Effects of temperature stress on intestinal tissues, immune response, intestinal microbiota, and inflammation in juvenile largemouth bass (*Micropterus salmoides*). Aquaculture.

[13] Feng J.P., Wei K.J., Xu J., Yu Y.Y., Xu B., Ma B.S., Zhu X.Y. (2021). Effect of glutathione on the growth and resistance to high temperature stress of *Procambarus clarkii*. Fresh. Water. Fish..

[14] Goikoetxea A., Sadoul B., Blondeau-Bidet E., Aerts J., Blanc M.O., Parrinello H., Barrachina C., Pratlong M., Geffroy B. (2021). Genetic pathways underpinning hormonal stress responses in fish exposed to short- and long-term warm ocean temperatures. Ecol. Indic..

[15] Hannan F.M., Leow M.K.S., Lee J.K.W., Kovats S., Elajnaf T., Kennedy S.H., Thakker R.V. (2024). Endocrine effects of heat exposure and relevance to climate change. Nat. Rev. Endocrinol..

[16] Shi K.P., Dong S.L., Zhou Y.G., Li Y., Gao Q.F., Sun D.J. (2019). RNA-seq reveals temporal differences in the transcriptome response to acute heat stress in the Atlantic salmon (*Salmo salar*). Comp. Biochem. Phys. Part D Genom. Proteom..

[17] Zhao H., Ke H., Zhang L., Zhao Z., Lai J., Zhou J., Huang Z., Li H., Du J., Li Q. (2022). Integrated analysis about the effects of heat stress on physiological responses and energy metabolism in *Gymnocypris chilianensis*. Sci. Total. Environ..

[18] Zhang T., Zhang L., Yin T., You J., Liu R., Huang Q., Shi L., Wang L., Liao T., Wang W. (2023). Recent understanding of stress response on muscle quality of fish: From the perspective of industrial chain. Trends Food Sci. Technol..

[19] Daskalova A. (2019). Farmed fish welfare: Stress, post-mortem muscle metabolism, and stress-related meat quality changes. Int. Aquat. Res..

[20] Liu Y., Liu J., Ye S., Bureau D.P., Liu H., Yin J., Mou Z., Lin H., Hao F. (2019). Global metabolic responses of the lenok (*Brachymystax lenok*) to thermal stress. Comp. Biochem. Phys. Part D Genom. Proteom..

[21] Schreck C.B., Tort L., Farrell A.P. (2016). The Concept of Stress in Fish-Biology of Stress in Fish.

[22] Duan Y., Li H., Li J., Bai S., Fu S., Zhou Y., Liu S., Li R., Liu H., Zhou C. (2024). Integrated transcriptome and 16S rDNA analyses reveal that acute heat stress induces intestinal damage in *Gymnocypris eckloni*. Front. Mar. Sci..

[23] Zhao C., Wang J., Ren W., Zheng S., Ren Y. (2024). Histological, immune, and intestine microbiota responses of the intestine of rainbow trout (*Oncorhynchus mykiss*) to high temperature stress. Aquaculture.

[24] Wen C., Wei S., Zong X., Wang Y., Jin M. (2021). Microbiota-gut-brain axis and nutritional strategy under heat stress. Anim. Nutr..

[25] Zheng J., Cao J., Mao Y., Su Y., Wang J. (2019). Comparative transcriptome analysis provides comprehensive insights into the heat stress response of *Marsupenaeus japonicus*. Aquaculture.

[26] Hussein G.H.G., Chen M., Qi P.P., Cui Q.K., Yu Y., Hu W.H., Tian Y., Fan Q.X., Gao Z.X., Feng M.W. (2020). Aquaculture industry development, annual price analysis and out-of-season spawning in largemouth bass *Micropterus salmoides*. Aquaculture.

[27] Khosa D., South J., Cuthbert R.N., Wasserman R.J., Weyl O.L.F. (2020). Temperature regime drives differential predatory performance in Largemouth Bass and Florida Bass. Environ. Biol. Fishes.

[28] Li H.Y., Xu W.J., Wu L.Y., Dong B., Jin J.J., Han D., Xie S.Q. (2021). Differential regulation of endoplasmic reticulum stress-induced autophagy and apoptosis in two strains of gibel carp (*Carassius gibelio*) exposed to acute waterborne cadmium. Aquat. Toxicol..

[29] Zhang Y., Zhang Y., Ke T., Shi B., Huang L., Dong Z., Guo M., Mugeni C.S., Zhu A., Wang L. (2022). Hydrolysates of whole forage-fish and Pacific krill are useful to reduce fish meal in practical diets for largemouth bass (*Micropterus salmoides*), and dietary fish hydrolysate suppresses expressions of intestinal oligopeptide transporter and taurine transporter genes. Aquac. Res..

[30] Gong Y., Yang F., Hu J., Liu C., Liu H., Han D., Jin J., Yang Y., Zhu X., Yi J. (2019). Effects of dietary yeast hydrolysate on the growth, antioxidant response, immune response and disease resistance of largemouth bass (*Micropterus salmoides*). Fish Shellfish. Immunol..

[31] Livak K.J., Schmittgen T.D. (2001). Analysis of relative gene expression data using real-time quantitative PCR and the 2^−ΔΔCT^ Method. Methods.

[32] Lv H.B., Ma Y., Hu C.T., Lin Q.Y., Yue J., Chen L.Q., Zhang M.L., Du Z.Y., Qiao F. (2021). The individual and combined effects of hypoxia and high-fat diet feeding on nutrient composition and flesh quality in Nile tilapia (*Oreochromis niloticus*). Food Chem..

[33] Chuah S.X.Y., Mancera Azamar K.M., Kolli T., Odabasi A.Z., Zhang B., Sims C., Goodrich-Schneider R., Porras A.M., Farzad R. (2025). Texture engineering in cell-based seafood: Insights from structural and compositional benchmarks. Food Res. Int..

[34] Ali K.W., Suhad H.M. (2025). The role of environmental stress in fish health: A review. GSC Biol. Pharm. Sci..

[35] Liu Y., Liu Z., Xing T., Li J., Zhang L., Jiang Y., Gao F. (2023). Insight on the meat quality and carbonylation profile of breast muscle of broilers in response to chronic heat stress: A proteomic research. Food Chem..

[36] Suliman G.M., Hussein E.O.S., Al-Owaimer A.N., Alhotan R.A., Al-Garadi M.A., Mahdi J.M.H., Ba-Awadh H.A., Qaid M.M., Swelum A.A. (2023). Betaine and nano-emulsified vegetable oil supplementation for improving carcass and meat quality characteristics of broiler chickens under heat stress conditions. Front. Vet. Sci..

[37] Warner R.D. (2017). The eating quality of meat—IV water-holding capacity and Juiciness. Lawrie’s Meat Science.

[38] Li Y., Fu B., Zhang J., Wang G., Gong W., Tian J., Li H., Zhang K., Xia Y., Li Z. (2023). Effects of heat stress on the chemical composition, oxidative stability, muscle metabolism, and meat quality of Nile tilapia (*Oreochromis niloticus*). Food Chem..

[39] Wu Y., You X., Sun W., Xiong G., Shi L., Qiao Y., Wu W., Li X., Wang J., Ding A. (2021). Insight into acute heat stress on meat qualities of rainbow trout (*Oncorhynchus mykiss*) during short-time transportation. Aquaculture.

[40] Choi Y.M., Kim B.C. (2009). Muscle fiber characteristics, myofibrillar protein isoforms, and meat quality. Livest. Sci..

[41] Cohen-Kaplan V., Livneh I., Avni N., Cohen-Rosenzweig C., Ciechanover A. (2016). The ubiquitin-proteasome system and autophagy: Coordinated and independent activities. Int. J. Biochem. Cell Biol..

[42] Hu A., Li T., Zhou H., Guo F., Wang Q., Zhang J. (2024). Water binding ability changes of different proteins during high-moisture extrusion. Food Hydrocoll..

[43] Zhang Z.Y., Jiang Z.Y., Lv H.B., Jin J.Y., Chen L.Q., Zhang M.L., Du Z.Y., Qiao F. (2021). Dietary aflatoxin impairs flesh quality through reducing nutritional value and changing myofiber characteristics in yellow catfish (*Pelteobagrus fulvidraco*). Anim. Feed. Sci. Technol..

[44] Cai W., Liu H., He L., Fu L., Han D., Zhu X., Jin J., Yang Y., Xie S. (2023). Exercise training combined with a high-fat diet improves the flesh flavour, texture and nutrition of gibel carp (*Carassius auratus gibelio*). Food Chem. X.

[45] Cappello T., Mauceri A., Corsaro C., Maisano M., Parrino V., Lo Paro G., Messina G., Fasulo S. (2013). Impact of environmental pollution on caged mussels *Mytilus galloprovincialis* using NMR-based metabolomics. Mar. Pollut. Bull..

[46] Huo D., Sun L., Zhang L., Ru X., Liu S., Yang H. (2019). Metabolome responses of the sea cucumber *Apostichopus japonicus* to multiple environmental stresses: Heat and hypoxia. Mar. Pollut. Bull..

[47] Digilio G., Sforzini S., Cassino C., Robotti E., Oliveri C., Marengo E., Musso D., Osella D., Viarengo A. (2016). Haemolymph from *Mytilus galloprovincialis*: Response to copper and temperature challenges studied by 1H-NMR metabonomics. Comp. Biochem. Physiol. C Toxicol. Pharmacol..

[48] Hao R., Wang Z., Yang C., Deng Y., Zheng Z., Wang Q., Du X. (2018). Metabolomic responses of juvenile pearl oyster *Pinctada maxima* to different growth performances. Aquaculture.

[49] Zhang C., Deng D., Wu Y., Song L., Geng J., Feng H., Jiang S., Zhang K., Cheng Y., Yin S. (2025). New insights into the neurophysiological effects of heat stress on the Chinese mitten crab (*Eriocheir sinensis*). J. Therm. Biol..

[50] Zhao M., You X., Wu Y., Wang L., Wu W., Shi L., Sun W., Xiong G. (2022). Acute heat stress during transportation deteriorated the qualities of rainbow trout (*Oncorhynchus mykiss*) fillets during chilling storage and its relief attempt by ascorbic acid. LWT.

[51] Rigoulet M., Bouchez C.L., Paumard P., Ransac S., Cuvellier S., Duvezin-Caubet S., Mazat J.P., Devin A. (2020). Cell energy metabolism: An update. Biochim. Biophys. Acta (BBA)-Bioenerg..

[52] Ouyang X., Wei L., Tong X., Jiang F., Nie Q., Dong W., Sun D. (2025). Metabolomic analysis of hepatopancreas revealed the metabolic responses of clam *Cyclina sinensis* towards high temperature stress. Aquac. Res..

[53] Jiang Y., Jiao H., Sun P., Yin F., Tang B. (2020). Metabolic response of *Scapharca subcrenata* to heat stress using GC/MS-based metabolomics. PeerJ.

[54] Morris S.M. (2009). Recent advances in arginine metabolism: Roles and regulation of the arginases. Br. J. Pharmacol..

[55] Nüse B., Holland T., Rauh M., Gerlach R.G., Mattner J. (2023). L-arginine metabolism as pivotal interface of mutual host–microbe interactions in the gut. Gut Microbes.

[56] Cardona M., Izquierdo D., Barat J.M., Fernández-Segovia I. (2023). Intrinsic and extrinsic attributes that influence choice of meat and meat products: Techniques used in their identification. Eur. Food Res. Technol..

[57] Liu C., Meng F., Tang X., Shi Y., Wang A., Gu Z., Pan Z. (2018). Comparison of nonvolatile taste active compounds of wild and cultured mud crab *Scylla paramamosain*. Fish. Sci..

[58] Peng L., Zhang L., Xiong S., You J., Liu R., Xu D., Huang Q., Ma H., Yin T. (2024). A comprehensive review of the mechanisms on fish stress affecting muscle qualities: Nutrition, physical properties, and flavor. Compr. Rev. Food Sci. Food Saf..

[59] Wang W., Zhou X., Liu Y. (2020). Characterization and evaluation of umami taste: A review. Trac-Trends Anal. Chem..

[60] Peng L., You J., Wang L., Xiong S., Huang Q., Yin T. (2022). Effect of respite time before liver transportation on muscle quality of Blunt Snout (Wuchang) Bream. Foods.

[61] Tymchuk W., Sakhrani D., Devlin R. (2009). Domestication causes large-scale effects on gene expression in rainbow trout: Analysis of muscle, liver and brain transcriptomes. Gen. Comp. Endocrinol..

[62] Nguyen T.A., Lin J.M.G., Marques A.S.M.C., Fottner M., Bauer L.G., Reicher A., Daum D., Scrofani L., Liu Y., Cheng C. (2025). A non-enzymatic role of Nudix hydrolase 5 in repressing purine de novo synthesis. Science.

[63] Speers-Roesch B., Sandblom E., Lau G.Y., Farrell A.P., Richards J.G. (2010). Effects of environmental hypoxia on cardiac energy metabolism and performance in tilapia. Am. J. Physiol. Regul. Integr. Comp. Physiol..

[64] Hardie D.G., Scott J.W., Pan D.A., Hudson E.R. (2003). Management of cellular energy by the AMP-activated protein kinase system. FEBS Lett..

[65] Singh S., Anand R. (2023). Diverse strategies adopted by nature for regulating purine biosynthesis via fine-tuning of purine metabolic enzymes. Curr. Opin. Chem. Biol..

[66] Zhao H., Chiaro C.R., Zhang L., Smith P.B., Chan C.Y., Pedley A.M., Pugh R.J., French J.B., Patterson A.D., Benkovic S.J. (2015). Quantitative analysis of purine nucleotides indicates that purinosomes increase de Novo purine biosynthesis. J. Biol. Chem..

[67] Li Y., Ye Z., Xiang J., Li S., Zheng Z., Li Y., Fang Y., Zhang X., Chen X., Xue D. (2025). Purine nucleotide metabolism response to drought stress in rice. Plant Growth Regul..

